# Comparative Outcomes of Apixaban and Acenocoumarol in Advanced Chronic Kidney Disease and Atrial Fibrillation: A Retrospective Observational Study

**DOI:** 10.3390/jcm14248860

**Published:** 2025-12-15

**Authors:** Ioana Livia Suliman, Liliana-Ana Tuta, Florin Gabriel Panculescu, Andreea Alexandru, Dragos Fasie, Bogdan Cimpineanu, Georgeta Camelia Cozaru, Stere Popescu, Florin-Daniel Enache, Iulian Manac, Tatiana Chisnoiu, Luana Alexandrescu, Ion Bordeianu

**Affiliations:** 1 Faculty of Medicine, “Ovidius” University of Constanta, 900470 Constanta, Romania; panculescu_i@yahoo.com (I.L.S.); alexandra_med16@yahoo.com (A.A.); cimpineanub@yahoo.com (B.C.); stelu_popescu@yahoo.com (S.P.); florin.enache@365.univ-ovidius.ro (F.-D.E.); iulian.manac@gmail.com (I.M.); tatiana_ceafcu@yahoo.com (T.C.); luana.alexandrescu@365.univ-ovidius.ro (L.A.); ion_bordeianu@hotmail.com (I.B.); 2 “Sfantul Apostol Andrei” Emergency County Clinical Hospital, 900591 Constanta, Romania; dragosfasie@yahoo.com; 3 Center for Research and Development of the Morphological and Genetic Studies of Malignant Pathology (CEDMOG), Ovidius University of Constanța, 900591 Constanta, Romania; drcozaru@yahoo.com; 4 Internal Medicine Department, Academy of Romanian Scientist, 3 Ilfov Street, 050044 Bucharest, Romania

**Keywords:** chronic kidney disease, atrial fibrillation, anticoagulation, dialysis, bleeding risk

## Abstract

**Background**: Anticoagulation in patients with advanced chronic kidney disease (CKD) and atrial fibrillation (AF) remains challenging due to the concurrent risks of thrombosis and bleeding driven by endothelial dysfunction, uremic inflammation, and impaired hemostasis. Evidence comparing vitamin K antagonists (VKAs) with direct oral anticoagulants (NOACs) in this high-risk population, particularly in dialysis, is still limited. **Methods**: We conducted a single-center, retrospective observational study including 93 patients with CKD stages 4–5 and AF treated between January 2021 and February 2025. Fifty patients received apixaban (2.5–5 mg twice daily), and forty-three received acenocoumarol with a target INR of 2.0–3.0. Thirty-eight patients (41%) were on maintenance hemodialysis. Demographics, comorbidities, and risk scores (CHA_2_DS_2_-VASc and HAS-BLED) were analyzed. Bleeding events were classified per ISTH criteria. Statistical comparisons used *t*-tests and χ^2^ tests, with *p* < 0.05 considered significant. **Results**: The mean age was 67.8 ± 9.1 years, and 51.6% were male. Major bleeding occurred in 9.7%, minor in 15.8%, and overdose-related bleeding in 10.0% of patients. The overall bleeding rate was significantly lower in the apixaban group (16.0%) than in the acenocoumarol group (53.5%; *p* = 0.01). No thromboembolic events were observed in either group. Dialysis patients had higher bleeding rates overall (13.2% vs. 7.4%), mainly among those on VKAs. The HAS-BLED score moderately correlated with bleeding incidence (*r* = 0.43, *p* < 0.01). **Conclusions**: Apixaban provided comparable thromboembolic protection with significantly fewer bleeding events than acenocoumarol, including in patients on dialysis. These findings support apixaban as a safer and more practical anticoagulant option in advanced CKD, consistent with its limited renal clearance and reduced influence on the inflammation–coagulation axis. Further multicenter prospective studies are warranted to validate these real-world results.

## 1. Introduction

Chronic kidney disease (CKD) represents a major global health concern, affecting nearly 10% of adults and contributing substantially to cardiovascular morbidity and mortality. The progressive decline in renal function is tightly linked to systemic inflammation, oxidative stress, and endothelial dysfunction—pathophysiological mechanisms that together enhance both thrombotic and bleeding tendencies in this population [1]. Among individuals with CKD, atrial fibrillation (AF) is the most frequent sustained arrhythmia, with its prevalence increasing from <10% in early CKD to more than 40% in end-stage renal disease (ESRD) and among patients undergoing dialysis [2].

Anticoagulation in CKD remains a persistent clinical dilemma. The dual risk of thrombosis and hemorrhage is amplified by uremic platelet dysfunction, vascular calcification, and altered drug metabolism. Vitamin K antagonists (VKAs), such as acenocoumarol or warfarin, have been traditional standards of care; however, they are characterized by narrow therapeutic windows, unstable INR control, and multiple interactions with diet and concomitant medications. Conversely, direct oral anticoagulants (NOACs)—particularly apixaban, with approximately 27% renal clearance—have demonstrated favorable pharmacokinetic and safety profiles even in patients with advanced CKD [3].

In clinical practice, currently approved oral anticoagulation options in advanced CKD include vitamin K antagonists (VKAs) such as warfarin or acenocoumarol, as well as selected non-vitamin K oral anticoagulants (NOACs), primarily apixaban, rivaroxaban, edoxaban and dabigatran. VKAs have long served as standard therapy, but they present several disadvantages in CKD, including a narrow therapeutic range, high interindividual variability, numerous food–drug interactions, accelerated vascular calcification, and the need for frequent INR monitoring, which can be challenging in patients with impaired renal function. By contrast, NOACs offer more predictable pharmacokinetics, fewer interactions, and fixed dosing, but their renal elimination differs significantly; therefore, their use in severe CKD or dialysis requires careful selection and cautious interpretation of clinical data. Apixaban, characterized by the lowest renal clearance among NOACs (~27%), has recently emerged as a promising alternative in advanced CKD; however, limited randomized evidence in this high-risk population underlines the need for additional real-world validation.

Despite increasing evidence supporting the use of NOACs, pivotal randomized controlled trials have largely excluded patients with an estimated glomerular filtration rate (eGFR) < 30 mL/min/1.73 m^2^ or those on dialysis, leaving clinicians dependent on real-world data. Furthermore, most existing studies have been conducted in Western Europe or North America, while data from Eastern European populations remain limited. In these regions, constrained healthcare infrastructure and less consistent INR monitoring further complicate VKA management [4].

This study aims to provide real-world evidence comparing the safety and efficacy of apixaban versus acenocoumarol in patients with advanced CKD (stages 4–5) and AF, including both dialysis and non-dialysis populations. By integrating clinical and pathophysiological perspectives, we sought to determine whether apixaban offers comparable thromboembolic protection with a lower risk of bleeding in a population characterized by inflammation-driven vascular instability.

## 2. Materials and Methods

### 2.1. Study Design and Population

This retrospective, observational, single-center study included 93 consecutive adult patients with stage 4 or 5 chronic kidney disease (CKD) and confirmed atrial fibrillation (AF), treated between January 2021 and February 2025 in the Department of Nephrology, “Sf. Apostol Andrei” Constanța County Emergency Hospital, Romania. All patients had received continuous oral anticoagulant therapy for at least six months prior to inclusion.

Patients were divided into two groups based on the type of anticoagulant therapy:

NOAC group (*n* = 50): Apixaban was administered orally at a dose of 2.5 mg or 5 mg twice daily, adjusted according to internationally accepted criteria (age ≥ 80 years, body weight ≤ 60 kg, and/or serum creatinine ≥ 1.5 mg/dL), in alignment with current ESC/EHRA guidance.

VKA group (*n* = 43): Acenocoumarol was administered orally and titrated to maintain a therapeutic INR range of 2.0–3.0, based on standardized dose adjustment and monitoring recommendations.

Among the entire cohort, 38 patients (41%) were undergoing chronic hemodialysis three times per week (CKD stage 5D), and 55 (59%) were non-dialysis CKD stages 4–5 [5].

Due to the retrospective design and exclusive use of existing electronic medical records, blinding of investigators or treatment allocation was not applicable. To minimize selection and information bias, data extraction and verification were independently conducted by researchers who were not involved in patient management.

### 2.2. Inclusion and Exclusion Criteria

Inclusion criteria:

- Age ≥ 18 years;
- CKD stages 4–5 (estimated glomerular filtration rate [eGFR] < 30 mL/min/1.73 m^2^, calculated using the CKD-EPI 2021 formula);
- Documented AF (paroxysmal, persistent, or permanent, confirmed by ECG or Holter monitoring);
- Minimum three months of uninterrupted oral anticoagulation.

Exclusion criteria:

- Acute kidney injury or rapidly progressive glomerulonephritis;
- Active malignancy or hematologic disease;
- Concomitant dual antiplatelet therapy;
- Recent surgery or trauma (<3 months);
- History of kidney transplantation or peritoneal dialysis;
- Hemoglobin < 7 g/dL;
- Hospitalization within 30 days for major infection;
- Missing or incomplete clinical data [6].

### 2.3. Data Collection and Clinical Parameters

Demographic and clinical data—including age, sex, comorbidities (hypertension, diabetes mellitus, ischemic heart disease, heart failure, prior stroke/TIA), dialysis status, and laboratory parameters (hemoglobin, serum creatinine, eGFR, liver enzymes)—were extracted from electronic medical records. The chronicity of kidney disease was documented according to nephrology medical records, with all patients having a confirmed diagnosis of advanced CKD (stage 4 or 5) for more than 12 months prior to inclusion, reflecting stable chronic renal impairment rather than acute decline.

Inflammatory and biochemical parameters collected for all patients included hemoglobin, serum albumin, C-reactive protein (CRP), ferritin, fibrinogen, serum creatinine and eGFR, reflecting both nutritional status and systemic inflammatory burden.

Stroke and bleeding risk were assessed using the CHA_2_DS_2_-VASc and HAS-BLED scores, respectively [7].

Bleeding events were classified according to the International Society on Thrombosis and Hemostasis (ISTH) definitions:

- Major bleeding: Intracranial, retroperitoneal, or gastrointestinal bleeding requiring transfusion or hospitalization.
- Clinically relevant non-major bleeding (minor): Overt bleeding not fulfilling major criteria.
- Overdose-related bleeding: Bleeding associated with supratherapeutic INR > 4.5 or a documented dosing error [8,9].

### 2.4. Statistical Analysis

Continuous variables were expressed as mean ± standard deviation (SD), and categorical variables as frequencies or percentages. Between-group comparisons were performed using the independent samples *t*-test for continuous variables and the Chi-square or Fisher’s exact test for categorical variables.

Correlations between HAS-BLED score and bleeding incidence were evaluated using Pearson’s correlation coefficient. Statistical significance was set at *p* < 0.05.

### 2.5. Ethical Considerations

The study protocol was reviewed and approved by the Ethics Committee of the Clinical Emergency Hospital of Constanța County (Decision No. 55437/25.08.2025) and conducted in accordance with the principles of the Declaration of Helsinki. Written informed consent was obtained from all participants prior to inclusion [10].

## 3. Results

### 3.1. Baseline Characteristics

A total of 93 patients with CKD stages 4–5 and atrial fibrillation (AF) were included, of whom 51.6% were male. The mean age was 67.8 ± 9.1 years (range: 45–88). Thirty-eight patients (41%) were on chronic hemodialysis, while fifty-five (59%) had advanced non-dialysis CKD.

Hypertension was highly prevalent (92%), followed by diabetes mellitus (47%), ischemic heart disease (39%), and heart failure (44%). No significant differences in baseline demographics or comorbidities were observed between the two anticoagulant groups (*p* > 0.05). (see Table 1) [11].

Table 1 Baseline demographics and comorbidities were well balanced across groups, with no statistically significant differences in age, sex, or comorbid disease burden.

**Figure 1:**
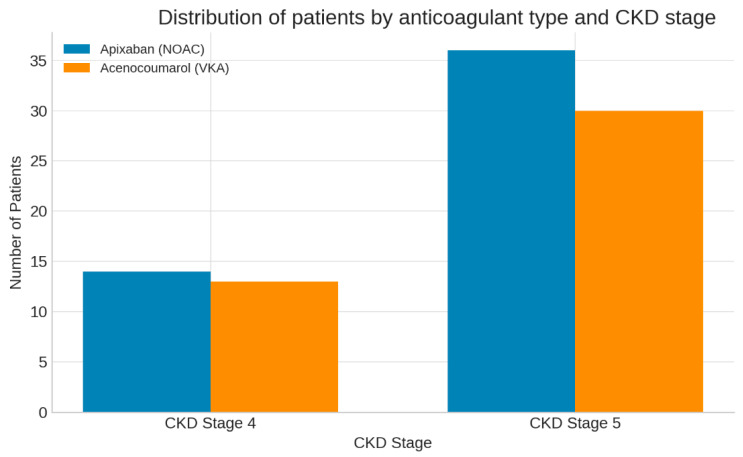
Distribution of patients by anticoagulant type and CKD stage. Bar chart showing the number of patients treated with apixaban (NOAC, blue) and acenocoumarol (VKA, orange) according to CKD stage 4 and 5. The two treatment groups were well balanced across renal function categories, confirming cohort comparability.

As shown in Figure 1, the distribution of patients by anticoagulant type and CKD stage was nearly identical, confirming group comparability.

### 3.2. Inflammatory and Biochemical Markers

Table 2 summarizes the comparative biochemical and inflammatory profiles of patients treated with apixaban versus acenocoumarol. Overall, apixaban users exhibited a more favorable biological pattern, consistent with reduced systemic inflammation and improved endothelial stability.

Mean hemoglobin levels were significantly higher in the apixaban group (11.2 ± 1.8 g/dL) compared with acenocoumarol users (10.4 ± 1.7 g/dL; *p* = 0.04). Similarly, serum albumin was modestly but significantly higher in apixaban-treated patients (3.8 ± 0.5 vs. 3.4 ± 0.6 g/dL; *p* = 0.02), reflecting better nutritional and inflammatory status. In contrast, C-reactive protein (CRP) concentrations were markedly elevated in the VKA group (13.7 ± 9.5 mg/L) compared with the apixaban group (8.9 ± 6.4 mg/L; *p* = 0.03), indicating a greater degree of oxidative–inflammatory activation among VKA users.

Although ferritin and fibrinogen levels were higher in patients receiving acenocoumarol, the differences did not reach statistical significance (*p* = 0.41 and *p* = 0.16, respectively). Serum creatinine was significantly greater in the VKA group (7.3 ± 2.8 mg/dL vs. 6.1 ± 2.3 mg/dL; *p* = 0.01), whereas estimated glomerular filtration rate (eGFR) values were comparable between groups (14.4 ± 7.8 vs. 12.9 ± 5.6 mL/min/1.73 m^2^; *p* = 0.27).

Correlation analysis revealed a moderate positive relationship between HAS-BLED and CRP (r = 0.42, *p* = 0.008), suggesting that systemic inflammation contributes to bleeding vulnerability in advanced CKD. Conversely, hemoglobin correlated inversely with HAS-BLED (r = −0.39, *p* = 0.01), indicating that anemia and inflammation coexist as synergistic risk factors for hemorrhagic complications.

Taken together, these biochemical findings support the hypothesis that apixaban may confer an anti-inflammatory and endothelial-stabilizing advantage, potentially mediated through selective factor Xa inhibition and reduced activation of protease-activated receptors (PAR-1/PAR-2) on vascular endothelium.

These differences suggest that patients receiving acenocoumarol may have had a more severe biological profile at baseline, which could partially contribute to their higher bleeding risk. This unmeasured confounding, inherent to observational designs, should be accounted for when interpreting comparative outcomes.

### 3.3. Bleeding Events

Over 18 months, 23 bleeding events were recorded (24.7% of the total population). Rates and severity differed significantly between treatments (see Table 3).

Table 3 Apixaban users had a significantly lower overall bleeding rate, driven primarily by fewer minor bleeding events.

Based on the ISTH definitions, bleeding events were categorized as major or clinically relevant minor hemorrhages. Patients treated with apixaban experienced fewer bleeding complications overall, with notably lower rates of both major and minor events compared with those treated with acenocoumarol. This difference aligns with challenges frequently encountered in real-world VKA therapy, including variability in anticoagulation control and a higher vulnerability to hemorrhagic complications in advanced CKD. These findings suggest a more favorable safety profile of apixaban in this population, while underscoring the importance of individualized anticoagulant selection in patients with impaired renal function [12].

**Figure 2:**
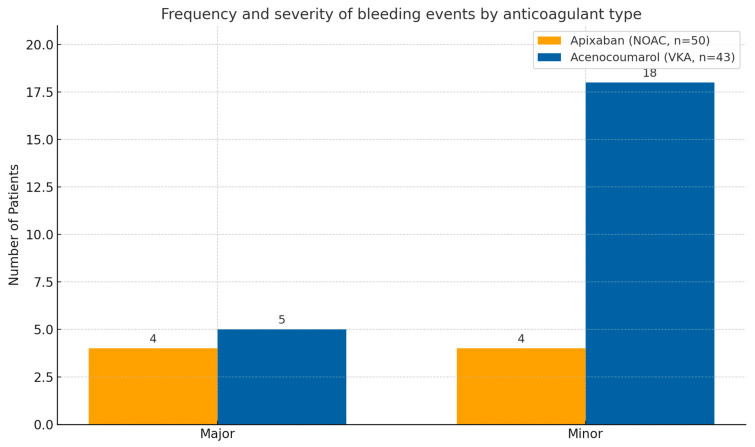
Frequency and severity of bleeding events by anticoagulant type. Comparison of major, minor bleeding events among apixaban (orange) and acenocoumarol (blue) users. Apixaban was associated with markedly fewer overall bleeding episodes.

Figure 2 illustrates the proportional distribution of major vs. minor events, clearly showing apixaban’s safety advantage.

### 3.4. Thromboembolic Events and Mortality

No thromboembolic events (stroke, systemic embolism, or transient ischemic attack) were observed in either group during the study.

All-cause mortality was low (5 patients, 5.3%) and slightly higher among VKA users (7% vs. 4%, *p* = 0.61) [13].

### 3.5. Correlation Between Risk Scores and Bleeding

Correlation analysis revealed a moderate positive association between HAS-BLED score and total bleeding events (r = 0.43, *p* < 0.01), confirming the predictive value of HAS-BLED in advanced CKD.

In contrast, CHADS-VASc showed no significant correlation (r = 0.12, *p* = 0.34), supporting the concept that thrombotic and hemorrhagic risks are largely independent [14].

### 3.6. Dialysis Versus Non-Dialysis Subgroups

Of the 93 patients, 38 (41%) were on maintenance hemodialysis (HD), while 55 (59%) were non-dialysis CKD 4–5.

Dialysis patients were slightly younger (66.1 ± 8.9 years vs. 69.5 ± 9.0 years, *p* = 0.09), had higher prevalence of heart failure (52% vs. 37%), and a greater frequency of anemia (Hb < 10 g/dL: 61% vs. 41%).

Event distribution between dialysis and non-dialysis patients is summarized in Table 4 [15].

Table 4 Dialysis patients exhibited higher bleeding frequencies overall, with overdose-related events confined to those treated with VKAs (*p* = 0.02).

**Figure 3:**
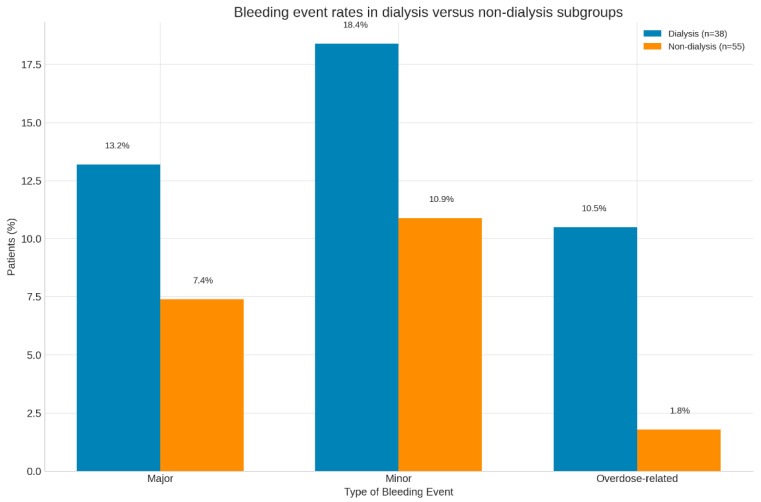
Bleeding event rates in dialysis versus non-dialysis subgroups. Percentage distribution of bleeding events by dialysis status. Dialysis patients (blue) experienced higher total and overdose-related bleeding rates than non-dialysis patients (orange), consistent with the increased fragility of ESRD populations.

Figure 3 presents contrast bleeding event proportions between dialysis and non-dialysis subgroups, highlighting the disproportionate risk among HD patients on VKAs.

## 4. Discussion

This retrospective observational study provides comparative evidence on the safety and efficacy of apixaban versus acenocoumarol in patients with advanced chronic kidney disease (CKD stages 4–5) and atrial fibrillation (AF), including both dialysis and non-dialysis populations. Our findings indicate that apixaban achieved comparable thromboembolic protection while significantly reducing bleeding events, especially minor ones, when compared with vitamin K antagonists (VKAs).

### 4.1. Principal Findings

Bleeding complications occurred in approximately one-quarter of the total cohort, reflecting the well-known hemorrhagic vulnerability of the CKD population. Apixaban use was associated with a 70% lower overall bleeding rate than acenocoumarol (16% vs. 53.5%, *p* = 0.01), while maintaining equivalent efficacy in preventing thromboembolic events. Because most bleeding events in our cohort were classified as minor, the clinical strength of this risk reduction should be considered with caution, particularly when extrapolating to severe bleeding outcomes.

Importantly, overdose-related hemorrhages occurred exclusively among VKA users, emphasizing the instability of INR control in patients with impaired renal function [16].

These findings are consistent with prior registry-based studies which demonstrated lower rates of major and clinically relevant bleeding in apixaban-treated ESRD patients compared with those receiving warfarin [17,18].

The pharmacokinetic profile of apixaban, characterized by limited renal clearance (~27%) and dual elimination pathways, supports its safety and predictable plasma levels even among dialysis patients [19].

Patients with advanced CKD are characterized by complex hemostatic derangements arising from both the uremic milieu and progressive endothelial dysfunction. Platelet abnormalities—including impaired adhesion, reduced glycoprotein IIb/IIIa expression, altered serotonin release, and decreased arachidonic acid signaling—contribute to the bleeding phenotype, whereas enhanced tissue factor release, increased thrombin generation, and downregulation of natural anticoagulants (protein C, protein S, and antithrombin III) promote a prothrombotic state. Concurrent anemia reduces blood viscosity and wall shear stress, further diminishing platelet–vessel wall interactions, while chronic inflammation accelerates vascular calcification and plaque instability, predisposing to thromboembolic events. Thus, CKD generates a protean interplay between thrombosis and hemorrhage in which standard coagulation markers insufficiently predict clinical risk, rendering anticoagulant therapy highly challenging and necessitating individualized drug selection.

### 4.2. Pathophysiological Insights

Advanced CKD promotes a complex imbalance between prothrombotic and hemorrhagic forces, often described as the “uremic bleeding paradox.” Uremic toxins impair platelet aggregation and adhesion, while chronic inflammation and oxidative stress enhance endothelial activation and tissue factor expression. Decreased nitric oxide bioavailability and vascular calcification further contribute to endothelial dysfunction and microvascular fragility [20].

Advanced CKD is characterized by simultaneous activation of coagulation pathways and impaired platelet function, reflecting a dysregulated hemostatic balance. Accumulation of uremic toxins, reduced nitric oxide bioavailability, and inflammation-mediated endothelial injury promote both thrombin generation and microvascular bleeding. Consequently, anticoagulation must be tailored to this fragile equilibrium to avoid tipping the balance toward harmful hemorrhage or thrombosis.

The association observed between apixaban therapy and a more favorable inflammatory and nutritional profile should be interpreted with caution. Given the retrospective nature of the present analysis, baseline inflammatory status prior to anticoagulation initiation was not uniformly available, and treatment allocation was not randomized. Therefore, channeling bias may have influenced prescribing decisions, with clinically more stable patients potentially being considered better candidates for NOAC therapy, whereas those exhibiting higher medical complexity or socio-economic vulnerability may have been preferentially initiated on VKAs. As a result, the differences in biomarkers likely reflect pre-existing patient characteristics rather than a direct anti-inflammatory effect of selective factor Xa inhibition. These findings should be regarded as associative signals, requiring confirmation in prospective studies specifically designed to evaluate biological pathways modulated by anticoagulants in advanced CKD [21].

From a pharmacological perspective, NOACs and VKAs exhibit distinct profiles that are relevant in CKD management. Apixaban shows predictable factor Xa inhibition and reduced reliance on renal clearance, with limited removal during hemodialysis. VKAs, in contrast, depend on hepatic metabolism, albumin binding, and vitamin K regulation, all of which may fluctuate significantly in CKD, especially in the presence of hypoalbuminemia or polypharmacy. These mechanistic distinctions provide a clinical background to understand real-world anticoagulation performance in severe renal impairment; however, they should not be interpreted as drivers of the outcomes observed in this study. Instead, the bleeding and biological findings presented herein indicate associated patterns that warrant further investigation through well-designed prospective studies focused on mechanistic interplay between anticoagulant therapy and coagulation biology in advanced CKD [22,23].

Beyond platelet dysfunction, the coagulation cascade in advanced CKD is profoundly altered. Increased circulating tissue factor and impaired clearance of activated coagulation factors promote excessive thrombin generation, whereas downregulation of natural anticoagulants such as protein C, protein S, and antithrombin III predisposes to a persistent prothrombotic state. Simultaneously, uremia-induced endothelial injury disrupts von Willebrand factor multimer distribution and nitric oxide signaling, further weakening primary hemostasis. Hemodialysis can exacerbate these abnormalities by inducing contact system activation and complement-mediated platelet consumption. Taken together, these mechanisms create a fragile and dynamic balance in which minor physiological shifts may abruptly tip the equilibrium toward hemorrhage or thrombosis, underscoring the need for anticoagulants with predictable pharmacokinetics in this population.

### 4.3. Dialysis Versus Non-Dialysis Patients

In our cohort, dialysis patients exhibited higher bleeding rates (13.2% vs. 7.4%), in line with previously reported ESRD data [24]. Hemodialysis inherently promotes platelet dysfunction due to repeated exposure to artificial membranes, systemic heparinization, and oxidative endothelial stress. Fluid shifts and altered protein binding further destabilize INR control during VKA therapy, leading to overdose-related hemorrhage.

The favorable safety profile observed with apixaban in our cohort was driven not only by fewer overdose-related events, as INR instability is inherently absent, but also by a lower incidence of clinically relevant non-major bleeding. The preservation of endothelial integrity and the avoidance of excessive anticoagulation likely contribute to this risk reduction. Furthermore, gastrointestinal bleeding—one of the most frequent complications in uremia due to angiodysplasia and mucosal friability—was substantially more prevalent among acenocoumarol users, possibly reflecting exacerbation of mucosal hypoxia by supratherapeutic INR. Importantly, the absence of documented thromboembolic events in both groups, despite high CHA_2_DS_2_-VASc scores, suggests that stroke prevention efficacy is retained with apixaban even in severe renal impairment. These results, in conjunction with emerging data from large US and European registries, support the applicability of apixaban as a safer anticoagulation strategy in ESRD, where the benefit–risk balance of VKAs has become increasingly questioned.

Apixaban’s minimal dialyzability (<5%) and stable pharmacokinetics under hemodialysis explain its superior safety profile in this subgroup [25]. Similar outcomes have been documented in large-scale studies demonstrating reduced bleeding and comparable stroke prevention with apixaban versus warfarin across CKD stages [26,27].

Although the baseline characteristics did not show statistically significant differences between groups, numerical imbalances in comorbidities such as diabetes mellitus and heart failure tended to favor the apixaban group. Additionally, the significantly higher serum creatinine values in VKA-treated patients despite similar eGFR may reflect lower muscle mass and a degree of nutritional frailty that is difficult to quantify retrospectively. These subtle but clinically relevant disparities could have contributed to the increased bleeding susceptibility and mortality in the acenocoumarol group. The potential influence of unmeasured confounders, inherent to any observational design, should therefore be acknowledged when interpreting comparative clinical outcomes.

Given that 41% of our cohort were undergoing maintenance hemodialysis, this subgroup represents a clinically relevant focus of the analysis. Hemodialysis exposes patients to additional bleeding risks through anticoagulation during dialysis sessions, platelet dysfunction induced by blood–membrane interaction, and higher inflammatory burden. These mechanisms likely contributed to the increased bleeding incidence observed among VKA-treated dialysis patients in our study, reinforcing the need for careful drug selection in this vulnerable population.

### 4.4. Clinical Relevance and Implications

Clinically, these results have practical implications, especially in healthcare systems where frequent INR monitoring is limited. Apixaban offers fixed dosing, fewer food or drug interactions, and predictable anticoagulant effects, translating into simplified long-term management and potentially better adherence [28].

From a public health standpoint, reduced bleeding-related hospitalizations may lead to cost savings and improved patient outcomes. These observations align with recent KDIGO and ESC recommendations that cautiously support NOAC use, particularly apixaban, in selected CKD stage 4–5 patients where the benefits of anticoagulation outweigh the bleeding risks [29,30].

From a translational perspective, these data support a paradigm shift in anticoagulation management for CKD and ESRD patients. Apixaban’s stable pharmacokinetics and reduced renal dependency make it a pragmatic and safer alternative to VKAs, particularly in environments where INR monitoring is inconsistent. Implementing NOAC therapy could decrease healthcare resource utilization and improve patient-reported outcomes.

### 4.5. Predictive Scores and Risk Stratification

The moderate positive correlation between HAS-BLED score and bleeding incidence (*r* = 0.43, *p* < 0.01) confirms the utility of this score in predicting hemorrhagic risk among patients with advanced CKD.

By contrast, the CHA_2_DS_2_-VASc score showed no significant relationship with bleeding (*r* = 0.12, *p* = 0.34), reinforcing that thrombotic and hemorrhagic risks in CKD are largely independent phenomena.

Incorporating renal-specific markers—such as albuminuria, high-sensitivity CRP, or endothelial biomarkers—into conventional scoring systems could enhance risk stratification and guide individualized anticoagulation strategies in the future.

### 4.6. Study Strengths

The study’s strengths include its real-world design, encompassing both dialysis and non-dialysis CKD patients, and detailed classification of bleeding severity using standardized ISTH criteria. Additionally, it represents one of the few Eastern European analyses directly comparing NOAC and VKA therapy in advanced CKD, thus expanding the geographic diversity of existing evidence.

### 4.7. Study Limitations

This study has several limitations. Its retrospective design introduces potential biases, including selection and reporting bias. The moderate sample size limits the ability to detect rare thromboembolic events or mortality differences. Furthermore, reliance on medical records may have underestimated minor bleeding episodes. Nonetheless, the observed trends are consistent with larger international datasets, supporting apixaban’s favorable safety profile in advanced CKD.

Although our study provides real-world evidence in a clinically challenging population, the overall sample size is modest, and the number of bleeding, thromboembolic, and mortality events remained low. As a result, the analysis may be underpowered to detect differences in rare outcomes, and subgroup comparisons should be considered exploratory.

All patients completed the full 18-month follow-up period, supported by continuous monitoring in a tertiary nephrology center and systematic verification of clinical events through electronic health records. The absence of stroke or systemic embolism and the relatively low mortality may reflect the benefits of rigorous anticoagulation management combined with vigilant surveillance typical of specialized care in advanced CKD. Nonetheless, we acknowledge that such outcomes must be interpreted with caution and confirmed in larger multicenter cohorts, where greater heterogeneity of clinical practice and case severity may yield different event rates.

Although the study was conducted within a single Eastern European center, the pathophysiological mechanisms governing anticoagulant response in CKD are universal and not restricted by ethnic or regional differences. Nevertheless, external validity should be interpreted in light of potential disparities in healthcare organization, access to dialysis modalities, nutritional patterns affecting vitamin K metabolism, and variability in INR monitoring infrastructure across global health systems. For these reasons, our findings warrant confirmation in multinational prospective cohorts incorporating diverse socioeconomic environments. Such validation would enable more definitive recommendations regarding optimal anticoagulant selection in advanced CKD and potentially inform the personalization of therapy based on renal phenotype, inflammatory burden, and pharmacogenetic background.

### 4.8. Future Directions

Future research should aim to validate these results in larger, multicenter prospective cohorts with longer follow-up durations.

Integrating molecular and inflammatory parameters—such as oxidative stress markers, endothelial function indices, and cytokine profiles—may help elucidate the biological mechanisms underlying the favorable safety of NOACs in renal impairment.

Moreover, assessing pharmacogenetic variability and its impact on apixaban metabolism could further personalize anticoagulation therapy.

Cost-effectiveness analyses and quality-of-life assessments will also be crucial for guiding clinical and policy decisions regarding NOAC implementation in ESRD populations.

## 5. Conclusions

In conclusion, in this retrospective single-center cohort of patients with CKD stages 4–5 and AF, apixaban was associated with a lower overall bleeding incidence compared with acenocoumarol, while maintaining similar thromboembolic protection, including among those on hemodialysis. However, these findings are primarily driven by reductions in minor bleeding, and the modest sample size and baseline imbalances limit the generalizability of treatment differences. Therefore, apixaban should be viewed as a potentially safer and more practical option rather than a definitive alternative to VKAs, pending confirmation in adequately powered prospective trials. Given the retrospective, single-center design and the relatively small sample size, these findings should be interpreted as hypothesis-generating rather than definitive evidence. Randomized multicenter studies are needed to confirm whether the observed safety advantages of apixaban can be generalized to broader advanced CKD populations.

## Figures and Tables

**Table 1:** Baseline clinical and demographic characteristics of patients.

Variable	Apixaban (*n* = 50)	Acenocoumarol (*n* = 43)	*p*-Value
Age (years)	68.6 ± 7.6	66.9 ± 10.6	0.47
Male (%)	52	49	0.74
eGFR (mL/min/1.73 m^2^)	14.1 ± 8.3	12.8 ± 5.9	0.55
CKD stage 5 (%)	72	70	0.81
Hypertension (%)	92	93	0.88
Diabetes mellitus (%)	44	51	0.49
Ischemic heart disease (%)	36	42	0.61
Heart failure (%)	40	49	0.42
Stroke/TIA history (%)	26	30	0.70
Hemodialysis (%)	40	42	*p* = 0.82

**Table 2:** Baseline biological and inflammatory markers according to anticoagulant type (*n* = 93).

Parameter	Apixaban (*n* = 50)	Acenocoumarol (*n* = 43)	*p*-Value
Hemoglobin (g/dL)	11.2 ± 1.8	10.4 ± 1.7	0.04 *
Serum Albumin (g/dL)	3.8 ± 0.5	3.4 ± 0.6	0.02 *
C-reactive protein (CRP, mg/L)	8.9 ± 6.4	13.7 ± 9.5	0.03 *
Serum Creatinine (mg/dL)	6.1 ± 2.3	7.3 ± 2.8	0.01 *
eGFR (mL/min/1.73 m^2^)	14.4 ± 7.8	12.9 ± 5.6	0.27
Ferritin (ng/mL)	325 ± 118	348 ± 132	0.41
Fibrinogen (mg/dL)	427 ± 91	458 ± 102	0.16
INR (VKA only)	—	2.8 ± 0.7	—
HAS-BLED score	5.3 ± 1.1	5.5 ± 1.0	0.56
CHA_2_DS_2_-VASc score	5.7 ± 1.0	5.9 ± 1.3	0.61

* Values are expressed as mean ± standard deviation. Statistical significance was defined as *p* < 0.05. Elevated CRP and reduced albumin levels reflect higher systemic inflammation in the VKA group, whereas apixaban-treated patients demonstrated more favorable oxidative-inflammatory profiles.

**Table 3:** Incidence and classification of bleeding events by anticoagulant type.

Event Type	Apixaban (*n* = 50)	Acenocoumarol (*n* = 43)	*p*
Major bleeding	4 (8.0%)	5 (11.6%)	0.64
Minor bleeding	4 (8.0%)	18 (41.9%)	0.04
Total bleeding events	7 (16.0%)	15 (53.5%)	0.02

**Table 4:** Bleeding event rates according to dialysis status.

Event	Dialysis (*n* = 38)	Non-Dialysis (*n* = 55)	*p*
Major bleeding (%)	13.2	7.4	0.31
Minor bleeding (%)	18.4	10.9	0.25
Overdose-related (%)	10.5	1.8	**0.02**
Thromboembolic (%)	0	0	–

## Data Availability

The data presented in this study are available upon request from the corresponding author.
